# Smartphone Addiction and Depression among Low-Income Boys since COVID-19: The Moderating Effect of Being an Only Child

**DOI:** 10.3390/healthcare9101350

**Published:** 2021-10-11

**Authors:** Jaewon Lee, Hyejung Lim, Jennifer Allen, Gyuhyun Choi, Jiyu Jung

**Affiliations:** 1 Department of Social Welfare, Inha University, Incheon 22212, Korea; j343@inha.ac.kr; 2 School of Education, Korea University, Seoul 02841, Korea; nanapro@korea.ac.kr; 3 School of Social Work, Michigan State University, East Lansing, MI 48823, USA; allenj66@msu.edu; 4 Integrative Arts Therapy, Dongduk Women’s University, Seoul 02748, Korea; toyou4048@nate.com; 5 Korea Development Bank Foundation, Seoul 07242, Korea

**Keywords:** smartphone addiction, depression, low-income, boys, being an only child

## Abstract

Even though boys’ depression has become important, and their smartphone use has increased since COVID-19, little is known about low-income middle and high school boys’ depression in the context of whether they have siblings. Thus, this study investigates the relationship between smartphone addiction and depression as well as the moderating effect of being an only child on the relationship. Participants were limited to middle and high school students whose families were regarded as having a low-income. A total of 129 low-income boys were selected for the final sample. The PROCESS macro 3.4 for Statistical Product and Service Solutions was used to identify the moderating effect. Smartphone addiction was positively related to depression among low-income male students. Being an only child significantly moderated the relationship between smartphone addiction and depression. This study contributes to understanding the importance of examining mental health problems among middle school boys since COVID-19, particularly among low-income boys. It is necessary to provide tailored mental health services for middle school boys in low-income families. Alternative activities and social programs should be provided for adolescent boys who are only children to safely socialize with friends and peers without a smartphone.

## 1. Introduction

The COVID-19 pandemic has greatly affected children’s and adolescents’ lives around the world. Compared to before the pandemic, children and adolescents have drastically increased their screentime, including their use of smartphones, and more children and adolescents are considered to be addicted to their smartphone or are engaging in problematic smartphone use than before the pandemic [1,2]. Moreover, children and adolescents are more depressed now than before the pandemic [3,4], and smartphone addiction and problematic smartphone use have been found to be positively associated with depression, although this data includes studies conducted before COVID-19 [5,6]. Researchers have also found that socioeconomic status may be associated with depression among children and adolescents, including during the COVID-19 pandemic [7,8], but other potential factors influencing smartphone addiction and depression, such as whether they have siblings to socialize with during periods of home confinement, have been understudied. Thus, in this study we examine the relationship between smartphone addiction and depression among adolescents in South Korea during the COVID-19 pandemic, and whether being an only child, or not having siblings, moderates the relationship.

### 1.1. Literature Review

#### 1.1.1. Smartphone Use among Adolescents since COVID-19

According to surveys administered to South Korean adolescents in 2018, at that time more than 95% of all middle and high school students had access to their own smartphone [9,10]. With modern adolescents in many countries having access to their own smartphone, it is of interest to examine their use of such devices. Further, since the COVID-19 pandemic and during associated lockdowns, research has found that adolescents’ use of smartphones has increased [1,11]. Among South Korean fourth graders, the percentage of children who used a smartphone more than 1 hour per day increased during the pandemic [1]. Additionally, among Italian youth aged 6–18 years, the percentage of youth who reported using a smartphone for more than 4 hours a day increased by 50% (from 16.3% to 66.3%) during the pandemic [11].

Other studies, rather than examining smartphone use specifically, examined an increase in screen time since the COVID-19 pandemic, including but not limited to smartphones [12,13,14]. Parents of children aged 10-18 years in Switzerland reported that their children had increased their media use significantly during the COVID-19-related lockdown, including their use of mobile phones, tablets, computers, video game consoles and televisions [14]. After the lockdown, most parents reported that their children’s media use returned to normal levels, but it remained higher among boys than girls [14]. Moreover, screen time increased by 4.85 hours per day among Italian children and adolescents three weeks into a COVID-19-related period of mandatory home confinement [12]. Last, compared to before COVID-19, German children and adolescents aged 4–17 years increased their recreational internet usage by 18.5 minutes per day and increased their total amount of recreational screentime by 61.2 minutes per day [13]. However, by examining screen time more generally and collapsing different types of technology use into a single variable, conceptual and empirical precision is decreased [15].

#### 1.1.2. Smartphone Addiction among Adolescents since COVID-19

Smartphone addiction is a common but growing issue among adolescents in South Korea [9], and studies in that country and others have shown that smartphone addiction and/or problematic smartphone use have increased among adolescents and adults since the COVID-19 pandemic [1,2,11,16,17]. South Korean fourth graders were more prone to smartphone addiction before COVID-19 than during [1]. However, problematic smartphone use prevalence was higher among Chinese primary schoolchildren during a COVID-19-related lockdown than before the pandemic [2]. Moreover, parents of American children aged 2–13 years reported that their children’s problematic media use had increased during the pandemic as compared to in Spring 2019 [16]. Rates of smartphone addiction also increased among Italian children and adolescents aged 6–18 years, with 31.5% of participants in one study at high risk of smartphone addiction and 26.1% with a documented smartphone addiction before the pandemic, compared to 27.2% of participants at high risk and 46.7% being addicted to their smartphone during the pandemic [11]. Smartphone addiction has also been found to be inversely associated with adolescents’ perceived socioeconomic status [18]. Similar to children and adolescents, problematic smartphone use was also found to increase during COVID-19 among undergraduates at a Spanish university, with mobile phone use increasing from being a risk behavior to a dependency behavior with the emergence of COVID-19 [17]. Thus, as evidence suggests smartphone addiction and problematic use has increased among multiple age groups since COVID-19, it is of interest to examine the relationship with other outcomes of interest, such as psychological well-being.

#### 1.1.3. Depression among Adolescents since COVID-19

Evidence suggests that the prevalence of depression among adolescents has increased since COVID-19 [3,4,19,20]. In a set of two surveys administered to Chinese adolescents, one in February 2020 and another in April 2020, the prevalence of depression in the sample rose by 20.4% at the second time point [19]. Moreover, between two surveys administered in February 2019 and June 2020, the clinical levels of anxiety and depression increased by 0.8% among the Norwegian youth surveyed, although the authors posit that this increase may be partially due to increases in age, rather than COVID-19 influences alone [20]. Depression also increased, particularly among girls, among Australian adolescents between a survey administered in the 12 months before the COVID-19 pandemic, and again two months following COVID-19-related Government-mandated restrictions and a transition to online learning [4]. Further, among a sample of Canadian adolescents who were surveyed at four time points prior to the pandemic and one time point during, the researchers used latent growth modeling, which demonstrated that adolescents’ depression scores during the pandemic were significantly higher than previous trajectories would be predicted [3]. Two studies also noted that depression was higher among adolescents who lived in high poverty areas or whose families had experienced new financial difficulties during the pandemic [7,8]. In a sample of adolescent athletes in the United States, adolescents who lived in a high poverty county exhibited the highest levels of depression, compared to those who lived in counties with a low or middle poverty level [7]. Additionally, in a sample of children in England during the pandemic, children with probable mental health conditions, which included but was not limited to depression, were more than twice as likely to reside in a household that had newly fallen into debt, indicating financial difficulties or hardship related to the pandemic and supporting the idea that adolescents in lower-income households or in poverty are more at risk for depression during COVID-19 [8].

## 2. The Current Study 

Smartphone addiction has been associated in some studies with increased depression among adolescents, including among those in low-income families [5,6,21,22,23]. Since COVID-19, middle and high school students have been less likely to meet their friends in person due to the risk of spreading coronavirus. Along with the reduced frequency of meeting with friends in person and the emergence of online classes since COVID-19, students may be more likely to become addicted to using their smartphone [1,2,11,16,17]. With reduced in person contact, they tend to be more isolated, leading to increased risk of depression. Depression among women or girls has been paid more attention in the literature, as they generally have higher rates of depression than men or boys [24,25]; thus, few studies have addressed depression among low-income boys. In person communication with others is important to prevent depression, so that only children, or children with no siblings, might be exposed to higher risk of depression since COVID-19, compared to those with siblings. As such, boys with no siblings might be at a higher risk of depression because they have limited resources which can be helpful to reduce depression due to COVID-19. Even though boys’ depression has become important since COVID-19 due to changed environments, little is known about low-income middle and high school boys’ depression in the context of whether they have siblings. Thus, this study investigates the relationship between smartphone addiction and depression among low-income boys as well as the moderating effect of being an only child on the relationship.

## 3. Methods

### 3.1. Participants and Sampling

Participants in this study include middle and high school students whose families were regarded as having a low-income. The yearly poverty threshold was considered to identify if families were considered to be a low-income household. Data collection was conducted in South Korea during April 2021. We used a nationwide sample of students who were enrolled in the Korea Development Bank [KDB] Foundation program, which is a non-profit organization. The foundation used a yearly poverty income guideline to select low-income students. A total of 264 middle and high school students joined the KDB Foundation’s program, and an online questionnaire was distributed to them. The research team used Google Forms to distribute the online survey. The questions in the survey were refined based on feedback and comments from a schoolteacher and social workers to reduce potential risks of the survey and protect students’ rights. The research team reached out to participants via text message to provide the survey link. A consent form was also provided to both students and their caregivers, so some students were excluded if either the students or their caregivers refused to engage in the online survey. Respondents who participated in the survey received a CAD 5 gift cards as an incentive to participate. As this study only focuses on boys, we excluded girls in this study. A total of 129 boys completed the online survey, and those who declined the survey or who we could not contact were not included in the final sample. A total of 42 boys were middle school students and 87 boys were high school students. This study does not include any private information such as name, address, etc. The Institutional Review Board approved the current study (#210216-2A).

### 3.2. Measures

#### 3.2.1. Depression

Depression in this study was measured by the Center for Epidemiologic Studies Depression Scale [CES-D]. We used a short version of the CES-D with seven items. The specific items included in the questionnaire are as follows: “I did not feel like eating; my appetite was poor;” “I had trouble keeping my mind on what I was doing;” “I felt depressed;” “I felt that everything I did was an effort;” “My sleep was restless;” “I felt sad;” and “I could not get going.” The CES-D measure has a four-point Likert-type scale, and “0 = rarely or none of the time, 1 = some or little of the time, 2 = moderately or much of the time, and 3 = most or almost all the time” were provided as response options. Each item was summed, with higher scores indicating higher levels of depression. The Cronbach’s α of this variable was 0.80 (Range = 0–21).

#### 3.2.2. Smartphone Addiction

Participants were asked to report to what extent they were addicted to using their smartphone. Smartphone addiction in this study refers to levels of students’ behavioral or psychological dependence on their smartphone. To measure their problematic smartphone use, five questions were given to students: “I have lost control managing the time I use my smartphone”, “I am not able to focus on other work due to my smartphone use”, “I am always thinking about using my smartphone”, “I have frequently fought with my family members due to my smartphone use”, and “I have difficulty in my school life, including academic performance”. The five items were rated on a five-point Likert-type scale with response options ranging from 1 (strongly disagree) to 5 (strongly agree). All items were summed, and higher scores indicate higher levels of smartphone addiction. Cronbach’s α of the smartphone addiction variable was 0.87 (Range = 5–25).

#### 3.2.3. Being an Only Child

Respondents were queried about how many siblings they have to determine whether they are an only child. Students who reported that they have no siblings were regarded as an only child (coded = 1), while those who have at least one sibling were considered as those with brothers or sisters (coded = 0). 

#### 3.2.4. Control Variables

Students’ age and academic achievement (i.e., letter grades of A, B, C, etc.) were included. Their parents’ educational attainment was also considered. If both their mother and father received higher than a high school education, they were regarded as having parents with higher education.

### 3.3. Analysis Strategies

**Figure 1:**
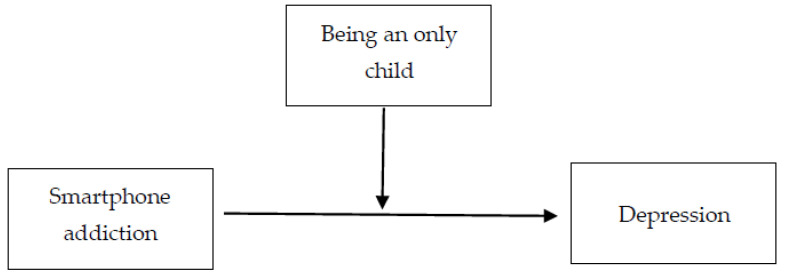
Research framework.

We used the PROCESS macro 3.4 for Statistical Product and Service Solutions (SPSS) (IBM, Armonk, NY, USA) to identify the moderating effect of being an only child on the relationship between smartphone addiction and depression amongst low-income, male middle and high school students. Model 1 was employed to test the moderating effect and a bootstrap method was used [26]. Figure 1 shows the research design of this study.

## 4. Results

Table 1 shows the descriptive statistics in this study. Respondents’ average scores of depression and smartphone addiction were 5.47 and 10.97, respectively. Approximately one-fourth of respondents were only children, while 74.4% had brothers or sisters. The average age of students in this study was 17.57 years old, but their international age is 16.57, because South Korea considers a newborn to be 1 year old. They further reported that their average academic performance was 7.67, which means a letter C grade in major classes. Further, 43.4% of students reported that both of their parents, including fathers and mothers, obtained higher education. 

**Figure 2:**
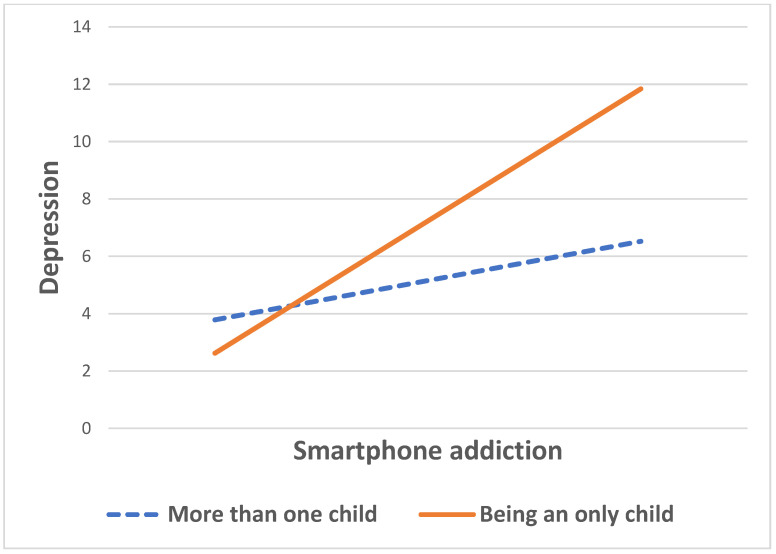
Moderating effect of being an only child on the relationship between smartphone addiction and depression among low-income boys.

Table 2 indicates the moderating effect of being an only child on the relationship between smartphone addiction and depression among low-income male students. Being an only child significantly moderated the relationship (β = 0.54, *p* < 0.01). In addition, smartphone addiction was positively related to depression (β = 0.23, *p* < 0.01), and age was also statistically associated with depression (β = 0.51, *p* < 0.05). For the moderating effect, specific information was provided in Figure 2. All students, regardless of whether they have siblings, were more likely to be depressed if they were more addicted to their smartphone. However, there was a gap in depression scores between those who had siblings and those who were only children. For students who were addicted to their smartphone, those with siblings showed relatively low levels of depression (6.52), compared to those without siblings (11.84). That is, there was a score difference of 5.32 between the two groups. On the other hand, there was a small gap in depression score between those with siblings and those without siblings if they did not show smartphone addiction (3.78 and 2.62, respectively). Thus, low-income students’ depression since COVID-19 in the context of smartphone addiction was greatly influenced by whether they have siblings.

## 5. Discussion

The current study identified the association between smartphone addiction and depression among low-income boys since COVID-19 and tested the moderating effect of being an only child on the relationship. Coronavirus emerged over one year ago and has greatly changed our life patterns. The negative effects of COVID-19 might be very detrimental to low-income families, particularly their children. That is, it is critical to understand boys’ depression since COVID-19, which has not been widely addressed compared to girls’ depression. This study found that middle and high school boys in low-income households who were addicted to their smartphone were at greater risk of depression than those without such an addiction. In addition, the moderating effect of being an only child was significantly impacted the relationship between smartphone addiction and depression among low-income boys. In other words, after the emergence of COVID-19, boys showing smartphone addiction without siblings experienced higher levels of depression compared to those who were addicted to their smartphone but had siblings.

This study demonstrated that middle and high school boys who were addicted to their smartphone were likely to be depressed. There have been a large body of studies showing the relationship between smartphone addiction and depression among adolescents [5,21,22]. However, little is known about middle and high school boys’ depression, particularly those from low-income households since COVID-19. The advent of the smartphone has discouraged students to play outdoor activities and has decreased the frequency of and quality of interactions when meeting up in person with their friends [27,28,29,30,31]. Boys tend to show more preference for outdoor activities than girls [32], including physical activities such as football or basketball. However, the emergence of COVID-19 has interrupted such activities and forced them to stay at home. As a result, they might use their smartphone more frequently for fun, as well as for education as online classes allow students to frequently use it. Given that middle and high school students are not mature and thus may be less able to control their smartphone usage, and because their smartphone use may have increased due to COVID-19-related online education, many students might become addicted to their smartphone, even if their parents could monitor and control their usage.

For low-income students, their parents might have fewer opportunities to work from home and stay with their children since COVID-19, because they may be more likely to have a job that requires them to work in person, or because the detrimental economic impact of COVID-19 has made it more difficult to earn a living and thus requires more time for work to maintain the same level of income as before COVID-19. That is, parents in low-income households may not have sufficient time to monitor their children’s smartphone use, leading to higher risk of smartphone addiction among low-income boys. This study revealed that smartphone addiction was associated with higher levels of depression among low-income boys since COVID-19. As little evidence is available on depression among middle and high school boys in low-income families since COVID-19, this study’s findings in shed light on future directions to reduce depression among low-income boys. Many mental health services are focused on adults rather than adolescents. However, as smartphone addiction has increased among middle and high school students since COVID-19 [33] and it is associated with depression [5], it is necessary to provide tailored mental health services for middle and high school boys in low-income families. Given the risk of spread of COVID-19, counselling in person or social gatherings for them might not be the best approach to improve mental health problems. Home visits from social workers, for example, financially supported by the Government or communities, should be encouraged to protect low-income boys from depression. Further, programs that involve outdoor activities, to encourage boys out of their houses and to start communicating with others while keeping appropriate social distance to prevent the spread of COVID-19, could be provided to reduce depression among middle and high school boys.

We found that being an only child moderated the relationship between smartphone addiction and depression among low-income middle and high school boys. Middle and high school boys without siblings were more likely to be at greater risk for depression if they were addicted to smartphone use. During the COVID-19 pandemic, middle and high school students have been forced to stay home more because they often took their classes online and were encouraged to do so to avoid coronavirus disease. As a result, they have decreased time to talk to their peers in person. In particular, for boys, they were not able to play sports or other games with their friends and had fewer opportunities for physical activities, which is one major way for boys to make friends and interact with peers [34,35]. However, middle and high school boys rarely have an outlet for in person communications with their peers since COVID-19, but if they have siblings, they have more chances to mutually communicate with their brothers or sisters in person, without the use of a smartphone. For boys who are only children, they must depend on smartphone to interact with their friends and peers at their age. However, such increased smartphone use, which may lead to smartphone addiction, is harmful to boys’ psychological health, as smartphone addiction may be associated with a sense of isolation or loneliness [36,37]. In other words, middle and high school boys who have no siblings and use their smartphone for communication due to loneliness are more likely to be depressed as they use it more.

Generally, low-income students tend to be at higher risk of depression compared to those who are not low-income [7,8]. Given such risk of psychological health among low-income boys, boys without siblings are further exposed to higher risk of depression because COVID-19 social distancing measures have made it more likely for middle and high school boys who do not have siblings to interact with at home to become addicted to smartphone use. For instance, low-income parents might not have sufficient time and resources to monitor their boys’ smartphone use, thus leaving boys in such families with more time to become addicted to their smartphone, which might be even more of an issue if they do not have a sibling for communication or mutual interaction in person. Particularly, given that adolescent boys tend to enjoy playing games using their smartphone [38], low-income boys who do not have siblings to communicate with and who have less monitoring from their parents are likely to be depressed. Before the emergence of COVID-19, little attention has been given to depression among middle and high school boys in comparison with other topics. This study contributes to understanding the importance of examining mental health problems among middle and high school boys since COVID-19, particularly among low-income boys.

## 6. Conclusions

As many adolescent boys use smartphones for purposes other than socializing (e.g., games, YouTube videos, etc.) [38], programs that encourage adolescent boys to engage in COVID-19-safe activities or hobbies should be encouraged, such as in-home activities such as reading, cooking, or art, or outside activities such as sports or running, depending on the boy’s interests. Such programs could be administered by schools, libraries, or other organizations in the community. By encouraging adolescent boys to participate in activities that do not include smartphone use, these boys may be less likely to develop a smartphone addiction, and thus have fewer symptoms of depression. Although these suggestions are given in the context of COVID-19, where one must be mindful of social distancing and the spread of coronavirus disease, they persist even once such measures are no longer needed, to encourage adolescent boys to participate in diverse activities, reduce the likelihood of smartphone addiction, and therefore depression. In addition, low-income boys without siblings should be particularly targeted for mental health services since COVID-19. In other words, due to increased smartphone use and addiction since COVID-19, alternative avenues to interact with their peers should be provided to adolescent boys who are only children and/or come from low-income households. Such alternative activities may include social programs or physical activities operated by community centers, to encourage adolescent boys who are only children to safely socialize with friends and peers without a smartphone.

Although this study sheds light on the significance of low-income boys’ depression, several limitations should be noted. First, accessibility to a smartphone and internet differs across countries, so findings are limited to middle and high school boys in South Korea. This study does not consider differences across countries. Thus, we suggest that future studies account for such social, economic, and cultural differences. Second, we used a self-report questionnaire, so participants might show social desirability bias. Third, criteria for what is considered a low-income household differs between countries, depending on level of economic development. Thus, interpretations about low-income might be different across countries. Fourth, this study only recruited students in low-income families. That is, it is not possible to show the differences between middle or high-income households and low-income households. Therefore, we suggest that future studies take into account how income differences influence the relationship between smartphone addiction and depression.

## Figures and Tables

**Table 1:** Descriptive Statistics.

Variables	% or Mean (SD)
Depression	5.47 (4.44)
Smartphone addiction	10.97 (4.92)
Being an only child	25.60 %
Age	17.57 (1.89)
Academic achievement	7.67 (3.78)
Parents’ educational attainment	43.4%

**Table 2:** Moderating Effects of Being an Only Child on Depression Using SPSS PROCESS.

Variables		
(Constant)	−6.04 (3.84)	
Smartphone addiction	0.23 (0.08)	**
Age	0.51 (0.19)	*
Academic achievement	−0.00 (0.09)	
Parents’ educational attainment	−0.65 (0.72)	
**Moderator**		
Being an only child	−3.86 (1.96)	
**Moderating effect**		
Smartphone addiction * Being an only child	0.54 (0.18)	**

Note. * *p* < 0.05. ** *p* < 0.01.

## Data Availability

The data presented in this study are available on request from the corresponding author. The data are not publicly available due to ethical reasons.
